# EPOR基因变异致原发性家族性和先天性红细胞增多症2例报告并文献复习

**DOI:** 10.3760/cma.j.cn121090-20250320-00144

**Published:** 2025-11

**Authors:** 道华 宁, 蒙 焦, 莉 覃, 清妍 高, 丽娟 潘, 士强 曲, 冰 李, 泽锋 徐, 青 冷, 志坚 肖, 铁军 秦

**Affiliations:** 1 中国医学科学院血液病医院（中国医学科学院血液学研究所），实验血液学国家重点实验室，国家血液系统疾病临床医学研究中心，细胞生态海河实验室，天津 300020 State Key Laboratory of Experimental Hematology, National Clinical Research Center for Blood Diseases, Haihe Laboratory of Cell Ecosystem, Institute of Hematology & Blood Diseases Hospital, Chinese Academy of Medical Sciences & Peking Union Medical College, Tianjin 300020, China; 2 鞍山市中心医院，鞍山 114000 Central Hospital of Anshan, Anshan 114000, China; 3 天津医学健康研究院，天津 301600 Tianjin Institutes of Health Science, Tianjin 301600, China

## Abstract

本文报道了2例原发性家族性和先天性红细胞增多症（PFCP）病例，并进行文献复习。PFCP是一种罕见的常染色体显性遗传性疾病，其发病机制为EPOR基因的功能获得性突变，导致红细胞增殖的负向调控缺失。2例患者均为年轻女性，表现为单纯红细胞增多，具有明确的家族史，并检测到EPOR基因c.1316G>A（p.W439*）截断突变。目前，PFCP尚无统一的治疗方案，临床实践中多参考真性红细胞增多症的治疗原则，主要目标是预防血栓并发症。本文结合现有文献，对PFCP的临床特征、EPOR基因突变及其致病机制进行了详细讨论，并提出诊断和治疗建议。

## Abstract (en)

In this paper, two cases of primary familial and congenital polycythemia（PFCP）were reported, and the literature was reviewed. PFCP is a rare autosomal dominant inherited disease caused by a gain-of-function mutation in the EPOR gene, resulting in a loss of negative regulation of erythrocyte proliferation. The two patients were young women with simple polycythemia and clear family history, and identified to carry the truncated mutation c.1316G>A（p.W439*）of EPOR gene. At present, there is no unified treatment plan for PFCP. Currently, there is no standardized treatment for PFCP; management primarily aligns with guidelines for polycythemia vera, focusing on preventing thrombotic complications. This article discusses the clinical features of PFCP, EPOR gene mutations, and their pathogenic mechanisms, while providing diagnostic and therapeutic recommendations based on existing literature.

原发性家族性和先天性红细胞增多症（Primary familial and congenital polycythemia, PFCP）是由促红细胞生成素受体（EPOR）发生基因突变导致的一种显性遗传性红细胞增多症。EPOR发生获得性功能异常基因突变，导致调节红细胞增殖信号转导时缺乏负调节机制，从而引起红细胞增多[1]。本文报道2例EPOR基因突变致PFCP患者，并对其临床特征、基因突变及其致病分子机制进行文献复习。

## 病例资料

例1，女，23岁，主因“发现红细胞增多7个月”于2024年6月就诊我科。患者于入院前7个月因呼吸困难就诊当地医院，发现红细胞增多［WBC 5.97×10^9^/L，HGB 184 g/L，红细胞压积（HCT）53.3％（正常参考值35％～45％），红细胞平均体积（MCV）91.5 fl（正常参考值82～100 fl），红细胞平均血红蛋白含量（MCH）31.6 pg（正常参考值27～34 pg），红细胞平均血红蛋白浓度（MCHC）345 g/L（正常参考值316～354 g/L），PLT 202×10^9^/L］。肝肾功能正常。血气分析正常。骨髓细胞形态学分类：增生活跃，粒系∶红系为2.46∶1，粒系比例46.4％，增生活跃，晚幼粒细胞比例增高，杆状核粒细胞比例减低，形态大致正常；红系比例18.8％，增生活跃，中晚幼红细胞比例正常，形态大致正常，成熟红细胞形态大致正常；全片见巨核细胞18个，分类18个，其中幼稚巨核细胞2个，颗粒巨核细胞10个，产板巨核细胞6个，血小板小簇可见。骨髓活检：增生大致正常（造血细胞面积70％），粒红比大致正常，粒系以中性中幼粒细胞及以下阶段为主，红系增生大致正常，以中晚幼红细胞为主，巨核细胞2～10个/骨小梁间，以分叶核巨核细胞为主，网状纤维染色MF-0级。染色体核型：46，XX[10]。BCR::ABL、JAK2、CALR、MPN基因突变阴性。促红细胞生成素（EPO）1.55 mIU/ml（正常参考值4.3～29.0 mIU/ml），考虑真性红细胞增多症。否认吸烟及服药史，否认肿瘤病史，患者父亲患有红细胞增多症（WBC 6.53×10^9^/L，HGB 210 g/L，HCT 61.8％，MCV 91.3 fl，MCH 30.9 pg，MCHC 339 g/L，PLT 271×10^9^/L），未诊治。患者入院查体：颜面及颈部轻微潮红，浅表淋巴结未触及、肝脾肋缘下未触及，双下肢无水肿。二代基因测序检测：EPOR c.1316G>A（p.w439*），区带19p13.2，序列NM_000121，位置exon8，为截断突变（图1），EPOR c.1316G>A的杂合突变家系验证来自父亲。目前患者聚乙二醇干扰素治疗中，血常规（治疗后，2025年2月14日）：WBC 5.2×10^9^/L，HGB 171 g/L，HCT 52.1％，MCV 89.1 fl，MCH 29.2 pg，MCHC 328 g/L，PLT 178×10^9^/L。患者及其父亲均无血栓栓塞事件发生。

**图1:**
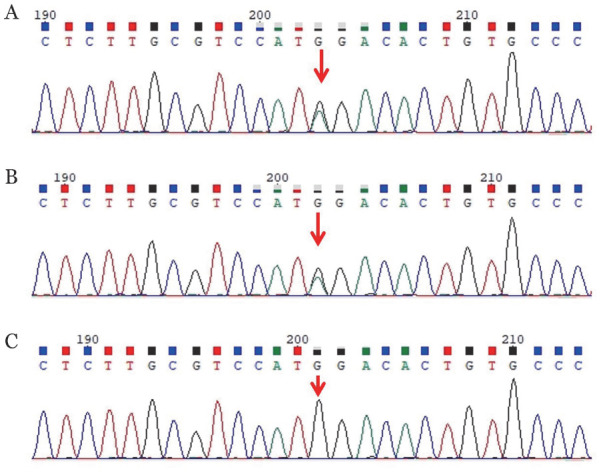
原发性家族性和先天性红细胞增多症患者（例1）及其父母EPOR基因测序图（箭头所示为突变位点） **A** 患者EPOR 19p13.2，NM_000121，exon8，c.1316G>A（p.w439*），为杂合突变；**B** 患者父亲EPOR 19p13.2，NM_000121，exon8，c.1316G>A（p.w439*），为杂合突变；**C** 患者母亲无基因突变

例2，女，21岁，主因“发现红细胞增多1个月”就诊我科。患者入院前1个月体检时发现HGB 200 g/L，无头晕、乏力、肢体麻木、活动障碍、腹痛、腹胀等不适。目前有资料可供分析的家系成员共7人，否认近亲结婚，谱系图见图2。入院查体：颜面及颈部潮红，口唇呈紫红色，浅表淋巴结未触及，肝脾肋缘下未触及，双下肢无水肿。血常规：WBC 10.67×10^9^/L，HGB 202 g/L，HCT 63.5％，MCV 90.6 fl，MCH 28.8 pg，MCHC 318 g/L，PLT 283×10^9^/L；EPO 6.46 mIU/ml（正常参考值2.59～18.5 mIU/ml）。肝胆脾超声：脾轻度肿大（长12.5 cm，厚2.6 cm，肋缘下0 cm）。骨髓细胞形态学：骨髓增生活跃，粒系∶红系为2.16∶1，粒系比例59.5％，以中晚幼及以下阶段粒细胞为主，形态大致正常；红系比例27.5％，增生活跃，以中晚幼红细胞为主，形态大致正常，成熟红细胞形态大致正常；全片见巨核细胞150个，分类25个，颗粒巨核细胞8个，产板巨核细胞16个，裸核巨核细胞1个，血小板成堆分布，易见。骨髓活检：增生活跃（60％～70％）；粒红比例大致正常，粒系各阶段细胞可见，以中幼及以下阶段细胞为主；红系各阶段细胞可见，以中晚幼红细胞为主；巨核细胞不少，分叶核细胞为主，可见胞体小、分叶少的巨核细胞；网状纤维染色MF-0级。染色体核型：46，XX[10]。BCR::ABL、JAK2、CALR、MPN基因突变阴性。二代测序检测：EPOR c.1316G>A（p.w439*），区带19p13.2，序列NM_000121，位置exon8，为截断突变。患者发病初期使用干扰素治疗，自2022年起，停用干扰素。截至2025年2月14日，其血红蛋白波动于160～202 g/L。正常生育一子。患者及其家系成员无血栓栓塞事件发生。

**图2:**
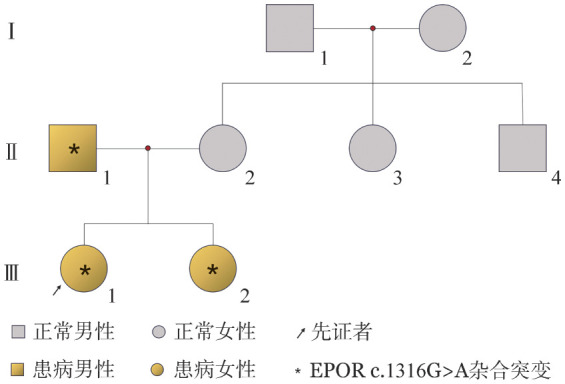
原发性家族性和先天性红细胞增多症患者（例2）家系图

先证者及家系成员HGB及HCT见表1；先证者父亲（Ⅱ-1）、姐姐（Ⅲ-2）同样存在HGB及HCT升高；先证者母亲（Ⅱ-2）、姨母（Ⅱ-3）、舅舅（Ⅱ-4）、外公（Ⅰ-1）及外婆（Ⅰ-2）的HGB及HCT正常。

**表1:** 原发性家族性和先天性红细胞增多症患者（例2）先证者及家系成员血常规血红蛋白及红细胞压积

成员	性别	年龄（岁）	HGB（g/L）	HCT（％）	MCV（fl）	MCH（pg）	MCHC（g/L）
Ⅰ-1	男	68	156	45.4	92.8	31.3	344
Ⅰ-2	女	67	130	40.8	90.2	31.6	319
Ⅱ-1	男	43	199	59.0	87.4	30.5	337
Ⅱ-2	女	43	129	39.0	88.3	30.9	331
Ⅱ-3	女	46	132	39.5	90.3	33.4	334
Ⅱ-4	男	41	159	47.5	88.9	30.3	335
Ⅲ-1	女	21	202	63.5	90.6	28.8	318
Ⅲ-2	女	23	162	51.0	87.6	26.6	318

正常参考值			110～150	37～48	80～100	27～34	320～360

**注** HCT：红细胞压积；MCV：平均红细胞体积；MCH：平均血红蛋白含量；MCHC：平均血红蛋白浓度

## 讨论及文献复习

先天性红细胞增多症（Erythrocytosis type/group，ECYT）共有8个类型（ECYT1～8）。其主要由九种基因通过不同机制介导，其中PFCP为先天性红细胞增多症1型（ECYT1），由EPOR基因突变导致。参与氧感知的基因突变（VHL、EGLN1、EPAS1、EPO）分别导致ECYT 2、3、4和5。影响血红蛋白氧亲和力的基因突变（HBB、HBA1和HBA2、BPGM）分别导致ECYT 6、7和8[2]–[3]。

人类EPOR基因定位于19p24，由EPO配体结合的外部结构域、一个跨膜结构域和一个内部结构域组成；包含8个外显子和7个内含子（508个氨基酸）。EPO结合EPOR导致EPOR发生同源二聚体构象改变，通过活化JAK2酪氨酸激酶，从而激活多种细胞内途径，包括Ras/MAP激酶、磷脂酰肌醇3激酶和STAT转录因子。EPO-R的C末端胞质结构域作为负调节结构域发挥作用，突变导致EPOR的C末端胞质负调节结构域酪氨酸残基丢失，负性调节JAK2活性的SHP-1、CIS、SOCS-3失去结合位点，导致JAK/STAT5等通路持续激活，引起红细胞增多。相关文献报道了33种EPOR基因变异与PFCP相关[4]–[6]，根据美国医学遗传学与基因组学学会和分子病理学协会（ACMG/AMP）指南，26种EPOR基因突变为致病或可能致病变异，3种为意义未明变异，4种为可能良性或良性变异。本文报道2例PFCP患者EPOR基因突变均为c.1316G>A（p.w439*）（位置exon8），为截断突变，属于致病性突变。

PFCP非常罕见，是一种常染色体显性遗传性疾病，迄今为止，文献仅报道了130余例。大多数患者无症状；部分患者因出现与红细胞增多相关的症状就诊，包括面色潮红、头痛、头晕、疲劳、全身无力、视力变化、精神模糊、耳鸣、胸痛、心悸、呼吸困难、腹部和骨痛、感觉异常、鼻出血和皮肤瘙痒等。个别病例出现心血管并发症和深静脉血栓形成。虽然临床表现类似于真性红细胞增多症，但一般没有进展为骨髓纤维化或转变为白血病的倾向[7]。1991年Juvonen等[8]报道了首例PFCP病例及其家系，患者为芬兰男性滑雪运动员（诊断时年龄53岁），自幼HGB保持在≥200 g/L水平，29岁时出现枕部出血，死于心肌梗死，享年76岁。包括患者的两个女儿在内29名家庭成员均有无症状性红细胞增多症。因此，当时的研究者认为PFCP对人类寿命并无影响。Prchal等[9]报道的1例PFCP患者发生了严重的心血管疾病，并在50岁时死于脑卒中，其同患红细胞增多症的儿子40岁时发生心肌梗死，这凸显了与EPOR突变相关的潜在严重血栓并发症。我们报道的2例PFCP患者均为年轻女性，主要临床表现为多血质症状及体征，患者及其家系成员均未发生血栓出血并发症。Gangat等[10]建议对于出现原因不明的孤立性红细胞增多患者，无脾肿大，血清EPO水平低于实验室参考范围，血红蛋白氧亲和力正常，且骨髓红系祖细胞对EPO敏感性增加时，考虑诊断PFCP。

PFCP作为罕见疾病，尚无共识性治疗路径，目前是多参考真性红细胞增多症的治疗方法。主要目标是预防血栓并发症。所有患者均应接受阿司匹林肠溶片治疗，以降低血栓形成风险。虽然静脉放血或单采红细胞有助于缓解症状，但似乎不能降低心血管事件风险[11]。也有学者认为大多数PFCP患者不需要治疗，仅部分患者应定期接受静脉放血治疗以控制红细胞压积、缓解高血黏度症状。对于35岁以上的患者动脉血栓发生率偏高，静脉放血或药物减细胞治疗似乎更为重要。儿童和青少年PFCP患者发生血栓形成的风险尚不清楚。儿童患者在HGB≤180 g/L时多无症状。有脑梗死、心肌梗死、脑出血家族病史的患儿，有可能从降低HCT治疗中获益[12]。本文报告的2例患者，例1接受了阿司匹林肠溶片和聚乙二醇干扰素治疗，血常规稳定；其父亲仅阿司匹林肠溶片预防血栓形成。例2及其家系患者均未接受阿司匹林肠溶片及减细胞治疗，均未发生血栓事件。英国血液学会最近建议采用静脉放血联合小剂量阿司匹林肠溶片治疗PFCP，并提出控制HCT目标值为小于52％[10]–[11]。

综上，本文国内首次报道了两例由EPOR基因c.1316G>A（p.W439*）截断突变导致的PFCP患者，并进行文献复习，对该疾病的临床特征、基因突变及其致病机制、治疗策略进行了初步探讨。PFCP是一种罕见的常染色体显性遗传性疾病，通常无血栓并发症，但存在并发血栓的潜在风险，以预防血栓形成为主要治疗目标。
